# Biological X-ray irradiator characterization for use with small animals and cells

**DOI:** 10.1590/1414-431X20165848

**Published:** 2017-03-02

**Authors:** A. Colello Bruno, S.J. Mazaro, L.L. Amaral, E.M. Rego, H.F. Oliveira, J.F. Pavoni

**Affiliations:** 1 Serviço de Radioterapia, Hospital das Clínicas, Faculdade de Medicina de Ribeirão Preto, Universidade de São Paulo, Ribeirão Preto, SP, Brasil; 2 Departamento de Física, Faculdade de Filosofia, Ciências e Letras de Ribeirão Preto, Universidade de São Paulo, Ribeirão Preto, SP, Brasil

**Keywords:** X-ray irradiator, Dosimetric characterization, Radiotherapy, Dosimetry, Radiochromic film

## Abstract

This study presents the characterization of an X-ray irradiator through dosimetric tests, which confirms the actual dose rate that small animals and cells will be exposed to during radiobiological experiments. We evaluated the linearity, consistency, repeatability, and dose distribution in the positions in which the animals or cells are placed during irradiation. In addition, we evaluated the performance of the X-ray tube (voltage and tube operating current), the radiometric survey (leakage radiation) and safety devices. The irradiator default setting was established as 160 kV and 25 mA. Tests showed that the dose rate was linear overtime (R^2^=1) and remained stable for long (constant) and short (repeatability) intervals between readings. The mean dose rate inside the animal cages was 1.27±0.06 Gy/min with a uniform beam of 95.40% (above the minimum threshold guaranteed by the manufacturer). The mean dose rate inside the cell plates was 0.92±0.19 Gy/min. The dose rate dependence with tube voltage and current presented a quadratic and linear relationship, respectively. There was no observed mechanical failure during evaluation of the irradiator safety devices and the radiometric survey obtained a maximum ambient equivalent dose rate of 0.26 mSv/h, which exempts it from the radiological protection requirements of the International Atomic Energy Agency. The irradiator characterization enables us to perform radiobiological experiments, and assists or even replaces traditional therapy equipment (e.g., linear accelerators) for cells and small animal irradiation, especially in early research stages.

## Introduction

Studies using small animals and cell experiments have become indispensable for cancer research before clinical implementation of a new therapy (1,2). They assist with the understanding of ionizing radiation interactions with tissues and cells, which is crucial for translational research of new effective radiotherapy techniques.

There are specific small animal irradiators designed especially for preclinical studies, used to evaluate and optimize new treatment modalities (3,4). Delineating set-up protocols with the equipment (linear accelerators) used clinically for patient treatments is a slow process, and using these irradiators for cells and small animal experiments in the preliminary stages of research would save time.

The most common irradiators used are the gamma-ray irradiators that employ radioactive isotopes such as cobalt-60 or cesium-137. However, recently, it has become increasingly difficult to purchase such irradiators because their manufacturing was interrupted. Additionally, the international transportation of isotopes involves radiation protection issues that complicate the process (5).

Thus, X-ray irradiators are an alternative for the gamma-ray irradiator and are being increasingly used due to their low cost and absence of a radioactive source (6,7). Other factors such as no facility-licensing requirements, and less rigorous and easier maintenance also add to the advantages of an X-ray unit (2,8).

For all ionizing radiation machines, certain quality assurance (QA) procedures are required to ensure basic operating conditions. However, there is no international QA recommendation for X-ray irradiators. One of the main goals of the QA procedures is to minimize errors related to dose delivery, which can be prevented using radiation detectors, such as ionization chambers, dosimetric films, or semiconductor detectors.

This study presents QA tests for X-ray irradiator characterization including dosimetric and safety tests, and a radiometric survey. Irradiator characterization is important for determining the dose distribution pattern and for evaluating the operating parameters in order to guarantee the dose deposition during irradiation. Both characteristics are essential for the quality of the translational research being developed.

## Material and Methods

This study was developed at the Radiotherapy Department of Ribeirão Preto Hospital and Clinics.

**Figure 1:**
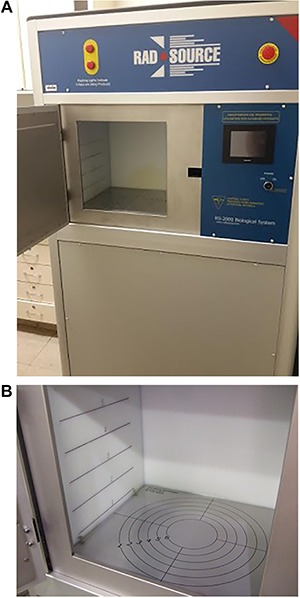
*A*, RS 2000 Irradiator. *B*, Irradiation chamber of the irradiator. Height levels for tray positioning (1 to 5), and the circles used for sample placement on the tray (1 to 6) are shown.

The X-ray irradiator (RS 2000 Biological System irradiator, Rad Source, USA) (Figure 1A) was characterized in order to establish the reference values for a QA program implementation in this machine. There is no international recommendation describing what tests should be applied or their frequency. We selected some tests to characterize this machine, evaluating its linearity, constancy, repeatability, dose distribution in the irradiation chamber, X-ray tube performance, in addition to safety test and radiometric survey.

The evaluated irradiator has six height levels available in its exposure chamber. A mobile tray with samples can be positioned at these levels and irradiated; therefore, six different dose rates can be achieved. On this tray, there are six circles that correspond to the size of the radiation field at a corresponding height (Figure 1B).

We chose the default position in the ionization chamber for film measurements, corresponding to the region inside circle 6 with the mobile tray at level 1 (Figure 1B). The default irradiation parameters for this irradiator were established at 160 kV (operating voltage) and 25 mA (operating current).

For the dosimetric characterization tests, we used an electrometer (Model Accu-Dose/2086, Radcal Corporation, USA), an ionization chamber (model 10X6-06-3, Radcal Corporation) and radiochromic films (Gafchromic EBT2, Ashland Advanced Materials, USA). A holder was used for positioning the ionizing camera on the region of interest. We also used a Thyac III Survey Meter (model 490, Victoreen Instrument Company, USA) for the radiometric leakage test.

### Linearity

Linearity is an important characteristic of the instrument that guarantees the equipment output. This is achieved when a specific change in the selected irradiation time generates a proportional change in the radiation generated. A linear relation between the irradiation time and the measured doses is expected.

To test the irradiator linearity, we measured the radiation doses produced for irradiation times varying from 0.5 to 10 min, using the ionization chamber located at the default position.

### Constancy

Constancy is another important characteristic as it is desirable that the equipment maintains the same output overtime. To evaluate the irradiator constancy, its dose rate or radiation output was measured at least once per month, for one year, with the ionization chamber in the default position. The acceptable variation of different readings was ±3% of the value obtained for the irradiator dosimetry. This follows the same criteria established for a medical linear accelerator by the American Association of Physicists in Medicine and Biology (9).

### Repeatability

Repeatability refers to the variation in measurements taken by a single person with the same instrument parameters, under the same conditions and within a short period of time; the agreement between the measurements guarantees that the instrument presents a precise output.

To evaluate the repeatability of dose measurements in the irradiator, seven dose measurements using a beam with a 1-min exposure were repeated with the ionization chamber in the default position.

### Dose distribution

The radiation dose distribution of any device is an important parameter to determine the absorbed dose distributions along the irradiated volume. The dose evaluation at any point on the irradiated volume considers the equipment dosimetry, usually performed at a reference point in the center of the radiation field, and by the radiation dose profiles, acquired in two orthogonal directions in the plane perpendicular to the radiation beam and in the vertical direction of the beam’s central axis.

Radiation dose profiles inside the exposure chamber from front to back (frontal) and left to right (lateral) were calculated. Radiation dose measurements were performed for 1 min using the ionization chamber; the chamber was moved by sliding the frontal and lateral axes in increments of 1 cm in the central region and 2 cm in peripheral regions. The tray was positioned at level 1 for all the measurements in order to characterize the largest field size available for irradiation.

Another profile was measured in the vertical direction inside the exposure chamber, and dose measurements were performed for 1 min in the beam central axis and varying the tray position from levels 1 to 5.

**Figure 2:**
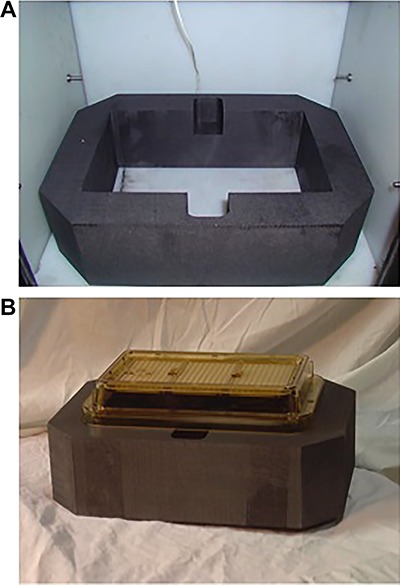
*A*, Shielded base and *B*, standard animal cage that fits perfectly inside the shielded base.

Small animals (e.g., mice and rats) are usually enclosed in a standard, acrylic animal cage during irradiation (Figure 2A and B). During this procedure, the tray is removed and the cage is placed on the floor of the exposure chamber, centered on a shielded base.

The animal cage fits perfectly inside the shielded base, which reduces stress in the animals and ensures that beam uniformity is greater than 95% on any horizontal plane. To evaluate the dose distribution inside the cage, we measured the average dose rate at five different points in the cage (at the four corners and at the center). Measurements were repeated with and without the cage cover (setup 1 and 2, respectively). Setup 1 was also repeated without the shielded base (setup 3).

The cell culture plate irradiation test was performed with samples positioned inside the circle corresponding to its respective tray level, as recommended by the manufacturer. To evaluate the dose distribution along the irradiation circle and inside cell culture plates, we used radiochromic films positioned in each of the six wells of the four culture plates that covered the entire irradiation circle. The cell culture plates were positioned in circle 1 with the tray on the first level (default setting for experiments with cells) and they were irradiated for 1 min.

### X-ray tube performance

The X-ray tube operating range varies from 30 to 160 kV, and its current varies from 5 to 25 mA; both are adjustable. The default irradiation parameters for this irradiator were established as 160 kV and 25 mA. It is known that these parameters directly influence the radiation output. In order to evaluate this influence, the dose rate per current was measured at all operating voltages, with the ionization chamber in the default position.

### Safety test and radiometric surveying

All safety devices were tested, including door interlocks, warning lights, emergency buttons, irradiation interruption, and apparatus intrinsic stability (warm-up). They were performed 72 times during an 18-month period. A radiometric survey was performed around the irradiator to evaluate the radiation levels in these areas. Equivalent dose rate measurements were monitored at 10 cm from each side of the irradiator (front, rear, right and left), using a Thyac III Survey Meter. At each side, three positions were measured: top, middle, and bottom.

## Results and Discussion

### Linearity

**Figure 3:**
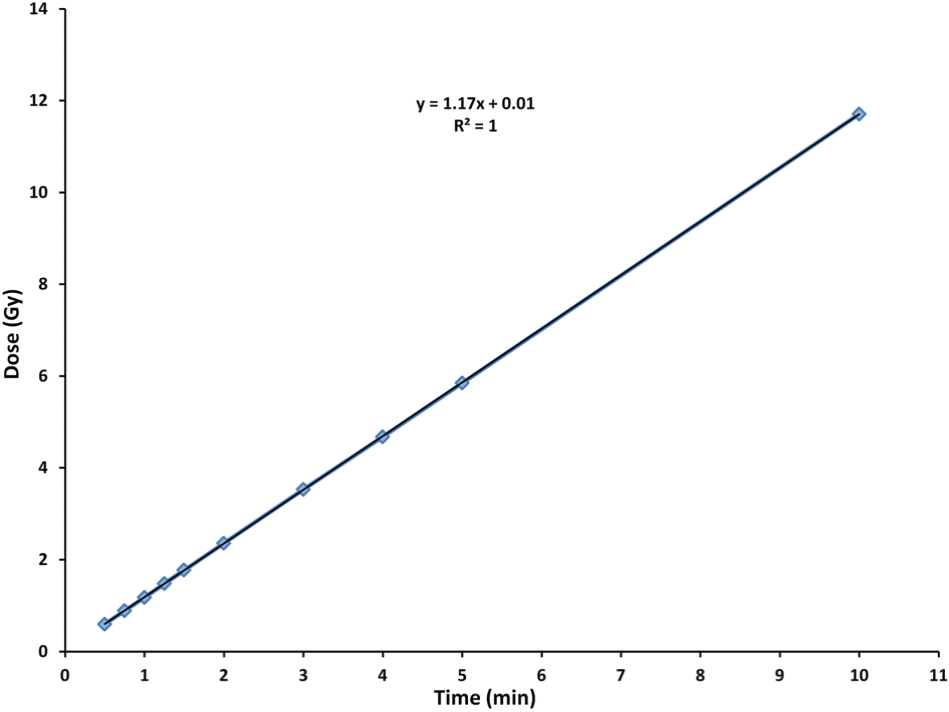
Irradiator linearity in the 0.5 to 10 min range.

The measured absorbed dose values for the respective irradiation time followed a linear pattern, indicated by the correlation coefficient (R^2^) equal to one (Figure 3). This result confirmed the expected linearity for irradiation times up to 10 min.

### Constancy

**Figure 4:**
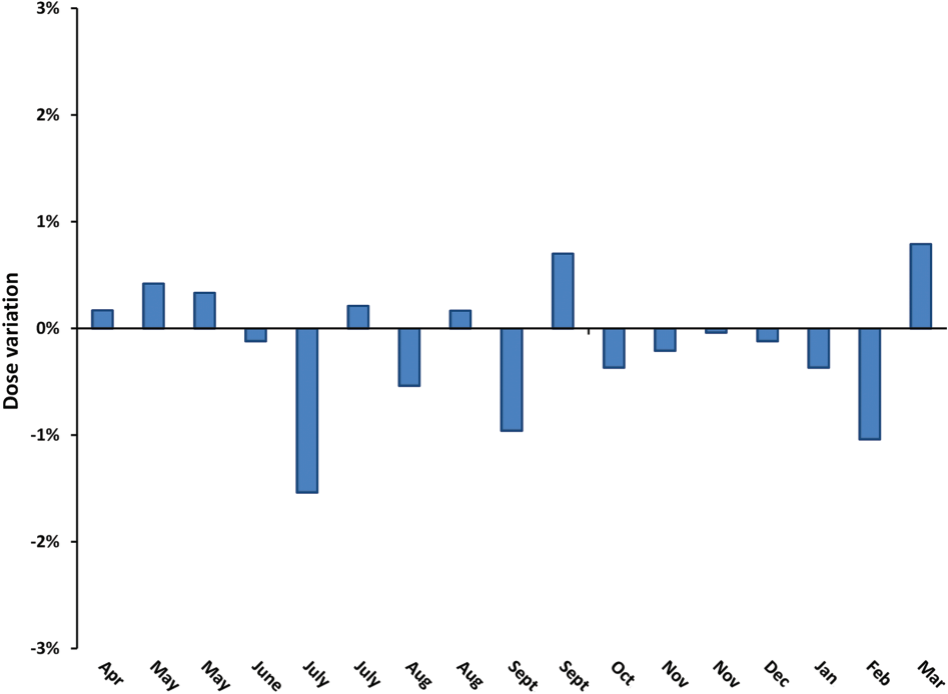
Variation of dose rates during 1 year. The data were obtained by the constancy evaluation in this period.

The dose rate variation during 1 year is presented in Figure 4. The maximum variation was 1.54%, which is below the ±3% acceptable limit.

### Repeatability

The ionization chamber’s readings for the repeatability study are shown in Table 1, including the mean value and the standard deviation. The values were very similar, with a standard deviation of 0.001 and a maximum deviation of 0.2%, confirming the equipment’s output precision.

**Table:**
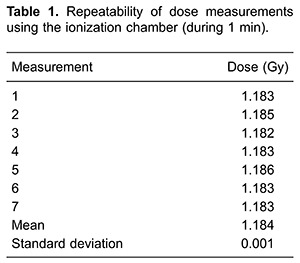

### Dose distribution

#### In exposure chamber

**Figure 5:**
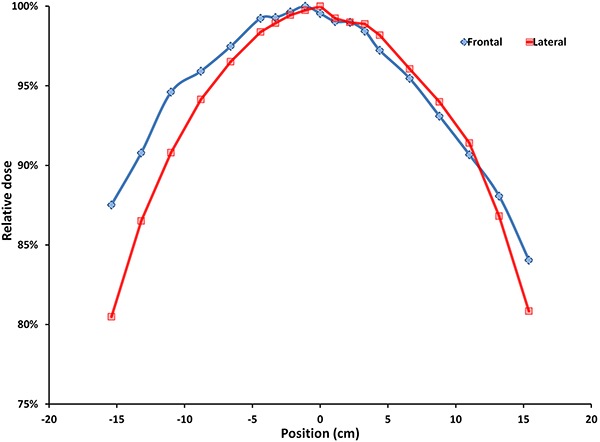
Dose distribution along the front and lateral axes of the exposure chamber, with the tray positioned at level 1.

**Figure 6:**
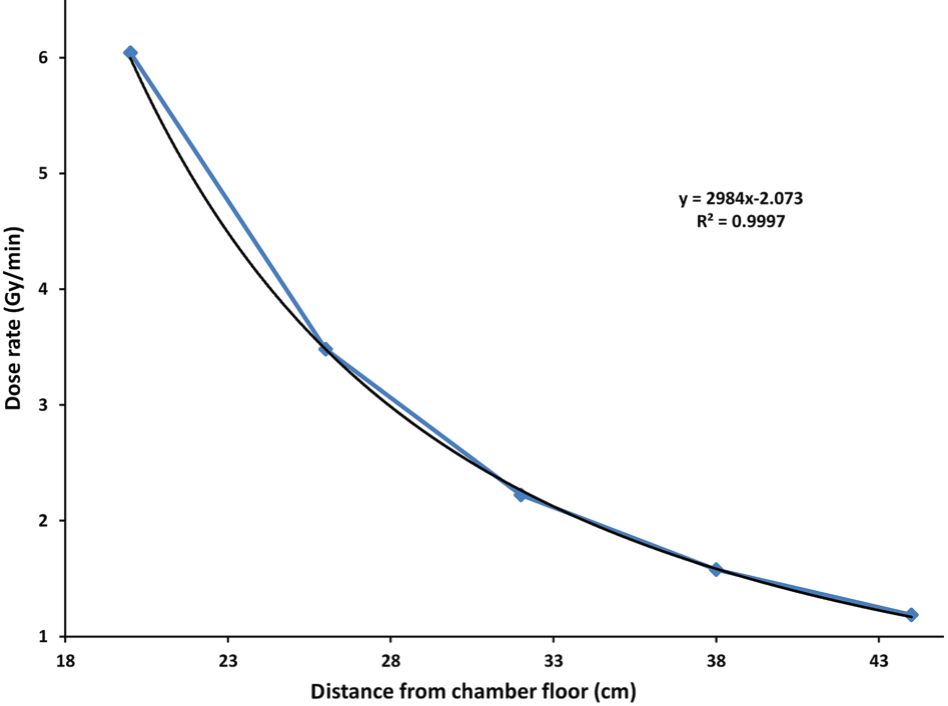
Dose distribution along the beam central axis varying the tray position from height levels 1 to 5.

Frontal and lateral dose profiles were achieved (Figure 5). Based on these data, it was possible to calculate the irradiation field symmetry, a parameter that describes the maximum percentage deviation between the doses at opposite sides of the central axis inside the flattened area (10). The symmetry for the frontal and lateral profiles was 3.47 and 0.35%, respectively. The asymmetry in the front axis was due to the anode heel effect, since the X-ray tube is positioned on this axial direction. The dose distribution along the beam central axis decreased following the inverse-square law, as the dose measurement point moves away from the radiation source (Figure 6).

#### In animal cages

The mean dose rate inside the standard animal cage measured for setups 1, 2, and 3 were 1.272±0.058, 1.343±0.041, and 0.989±0.07 Gy/min, respectively. For setups 1 and 2, the beam uniformity was higher than 95% (95.4 and 96.9%, respectively). However, for setup 3 this value was 92.7%. These values confirm the manufacturer information that the shielded base homogenizes the beam on the horizontal plane within the animal cage. It was also possible to determine that the cage cover attenuates 5.3% of the beam, a significant attenuation that should be considered during animal irradiation procedures.

#### In cell plates

The mean dose rate measured along circle 1, at the default setting for experiments with cells, was 0.917±0.189 Gy/min, with standard deviation corresponding to 20.6% of the dose rate value. However, with a repetition of this measurement covering irradiation circle 3, a smaller standard deviation was found: the mean dose rate was 1.117±0.062 Gy/min, with the standard deviation corresponding to 5.5% of the dose rate value.

The main reason for these high variations can be attributed to the cell plate materials, especially the cover, which attenuates the beam. This attenuation can be enhanced by increasing the distance from the irradiation circle center, increasing in the beam’s incidence angle on the plates.

### X-ray tube performance

**Figure 7:**
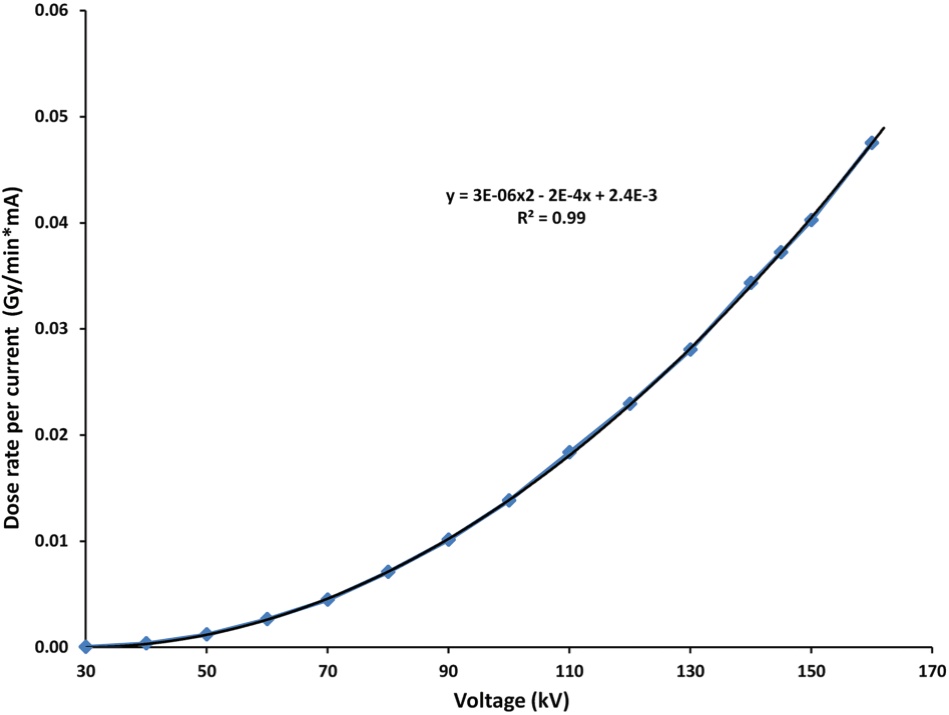
Relationship between dose rate and tube voltage (default position).

**Figure 8:**
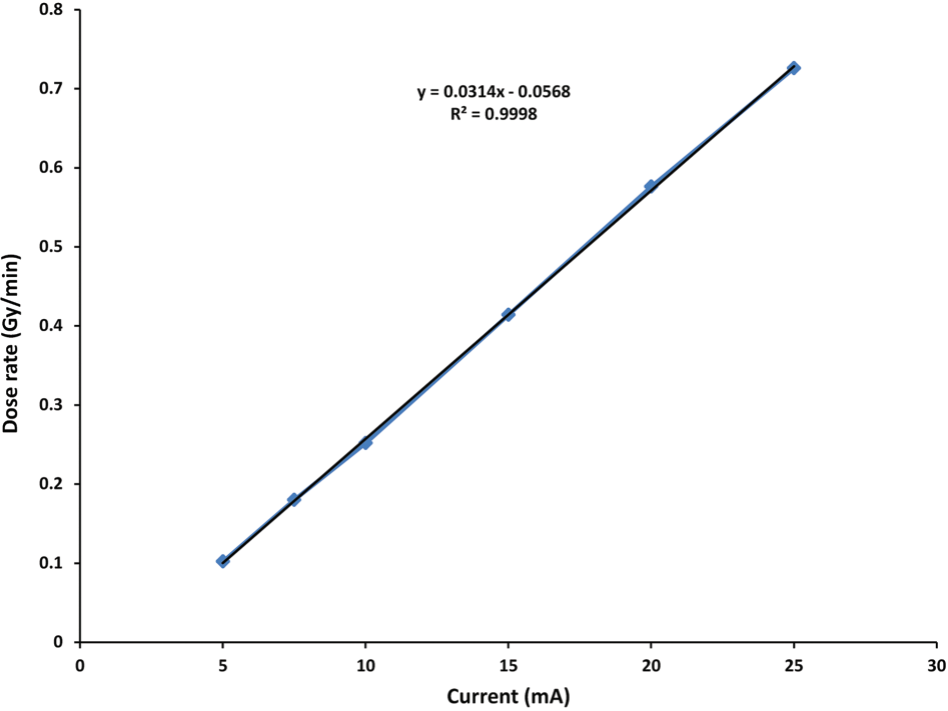
Relationship between dose rate and tube current (default position).

The dependence of the dose rate on the tube voltage was evaluated at the default position. As expected, it showed a good quadratic relationship (Figure 7), whereas dose rate was linearly dependent on the tube current (Figure 8).

### Safety test and radiometric surveying

There was no observed mechanical failure in the evaluation of the irradiator safety devices.

**Figure 9:**
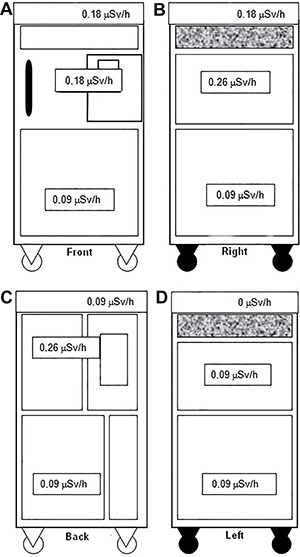
Experimental scheme for the irradiator radiometric survey. *A*, Front; *B*, right; *C*, back; and *D*, left.

Figure 9 presents the radiometric survey results with the equivalent dose rate values at the various measured points. All the values were below the minimum limit set by the International Atomic Energy Agency, which is 1 µSv/h at 10 cm around the equipment (11).

The reference values for a quality control program to be implemented in the X-ray irradiator were achieved by performing linearity, constancy, repeatability, dose distribution, and X-ray tube performance and safety tests. Special measurements were applied to evaluate dose distributions and their deviations for cells and small animal settings. The characterization of this biological X-ray irradiator enables us to perform radiobiological experiments, in order to assist, or even replace traditional therapy equipment (e.g., linear accelerators) for cells and small animal irradiation, especially in the early research stages.
